# The Latest South American Liner

**Published:** 1906-10-13

**Authors:** 


### THE LATEST SOUTH AMERICAN LINER.

### THE NEW TWIN-SCREW MAIL STEAMER "ARAGUAYA."

On Saturday last, October 6, the directors of the Royal Mail Steam Packet Company invited a large number of visitors to inspect the new steamer *Araguaya*, which is to run between England and South America. The *Araguaya* is the largest steamer in the R.M.S.P. Company's fleet, with a gross tonnage measurement of 10,537 tons. The decorations are of the most sumptuous kind and the entrance hall and dining saloon for first-class passengers display these to the best advantage. The former is panelled in oak picked out with gold, while the latter is brilliant with white and gold mouldings, giving in this way an air of cheerfulness and comfort.

In this new ship the directors of the company have professedly aimed at securing the best possible ventilation even during the hottest weather. With this end in view electric fans have been fitted at short intervals across the ceiling of the dining-room, but owing to their carefully chosen colour they are hardly apparent to any but an attentive observer. The dining saloon and the entrance hall above it are surmounted by a dome, the decorations of which are of the most lavish kind. Fresh air is forced through the whole ship from end to end at an equal pressure, which thus, while removing any impurities, at the same time insures against draughts. The sanitary arrangements appear perfect, there being a most liberal supply of bath-rooms and lavatories throughout the ship, which remind one far more of the luxury of a modern hotel than of the necessary economy of space which must always be a feature of maritime travel.

Of the delightful cabines de luxe, charming suites as they are, of the state cabins, and indeed of all the luxury that has been provided for what may be called the first-class part of the ship, sufficient notice has been taken in other quarters. We believe our readers will be more interested in the ship as regarded from the point of view of medical science and modern hygienic requirements. In these respects every class of passenger seems to have been considered, and we were especially glad to notice that particular arrangements had been made for the comfort of the children on board. For them a nursery has been provided, placed close to the first-class saloon, and in this way within easy reach of the parents. Besides this, in a central position are the doctor's consulting-room and dispensary, and the barber's shop, with a dark room for photographers.

A steam laundry has been fitted on the after deck, which, it may be noticed, is one of the few appliances on board not worked by electricity. A miniature hospital completes the list of advantages which care and foresight have provided through every branch of the ship's administration.

The *Araguaya* sails for South America on her maiden voyage next Saturday, the 13th inst., and from this description it will be seen that no pains have been spared to make her passengers comfortable and secure.

